# Transcriptome analysis of mouse aortae reveals multiple novel pathways regulated by aging

**DOI:** 10.18632/aging.103652

**Published:** 2020-08-15

**Authors:** Ping Gao, Pan Gao, Mihyun Choi, Kavya Chegireddy, Orazio J. Slivano, Jinjing Zhao, Wei Zhang, Xiaochun Long

**Affiliations:** 1 Department of Molecular and Cellular Physiology, Albany Medical College, Albany, NY 12208, USA; 2 Vascular Biology Center, Medical College of Georgia at Augusta University, Augusta, GA 30912, USA; 3 School of Public Health, University at Albany, Albany, NY 12222, USA

**Keywords:** transcriptome, arterial aging, protein folding control, circadian clock, vascular smooth muscle differentiation

## Abstract

Vascular aging has been documented as a vital process leading to arterial dysfunction and age-related cardiovascular and cerebrovascular diseases. However, our understanding of the molecular underpinnings of age-related phenotypes in the vascular system is incomplete. Here we performed bulk RNA sequencing in young and old mouse aortae to elucidate age-associated changes in the transcriptome. Results showed that the majority of upregulated pathways in aged aortae relate to immune response, including inflammation activation, apoptotic clearance, and phagocytosis. The top downregulated pathway in aged aortae was extracellular matrix organization. Additionally, protein folding control and stress response pathways were downregulated in the aged vessels, with an array of downregulated genes encoding heat shock proteins (HSPs). We also found that circadian core clock genes were differentially expressed in young versus old aortae. Finally, transcriptome analysis combined with protein expression examination and smooth muscle cell (SMC) lineage tracing revealed that SMCs in aged aortae retained the differentiated phenotype, with an insignificant decrease in SMC marker gene expression. Our results therefore unveiled critical pathways regulated by arterial aging in mice, which will provide important insight into strategies to defy vascular aging and age-associated vascular diseases.

## INTRODUCTION

Aging is a prominent risk factor for the development of cardiovascular diseases (CVDs), which account for one third of all deaths in the United States by the age of 65 and two thirds by age 85 [1]. Multifactorial cellular and molecular changes contribute to the deterioration of structure and function of the vascular tree during aging, such as decreased compliance and resilience, increased stiffness [2], luminal dilatation accompanied with arterial wall thickening, and reorganization of the extracellular matrix (ECM) [3]. These age-associated alterations render the vasculature susceptible to traditional risk factors (e.g. hypertension, diabetes, smoking) and prone to CVDs [4].

Vascular cells exhibit intrinsic age-related adaptations, including DNA damage and mutation, mitochondrial dysfunction, impaired proteostasis and autophagic activity, cellular senescence, as well as chronic low-grade inflammation [1, 5]. In particular, circadian rhythms, regulated by internal circadian clocks which are entrained to the environmental 24-hour day-night cycle, are critical for a healthy cardiovascular system. Various studies have shown that disruption of circadian rhythms in mice contributes to the development of age-associated CVDs [6–9]. Extrinsic changes such as vascular stiffness, chronic inflammation and alteration of the communication between endothelial cells (ECs) and vascular smooth muscle cells (VSMCs) have also been documented. These cell-autonomous and environmental mechanisms might be interrelated and act in concert to induce age-related vascular pathologies [10]. However, it remains unclear how the involved key pathways coordinately participate in vascular aging and age-associated vascular diseases.

Among numerous structural and functional alterations in aged large arteries, increased EC dysfunction and arterial stiffness have been defined as the most potent predictors for future CVDs in humans [5, 10]. These two risk factors are mutually exacerbating, rendering the etiologies of arterial aging and age-associated vascular diseases particularly complex. With age, EC dysfunction, presumably resulting from impairment in flow and reduced production of vasodilators, occurs leading to the dysregulation of vascular tone and loss in EC-dependent dilating capability [5, 11, 12]. This age-associated EC dysfunction correlates with the imbalance between vasodilator and vasoconstriction substances, both of which are produced by the endothelium [13]. On the other side, current concept of arterial stiffness has been extended to stiffness in both ECM and vascular cells, mainly VSMCs. Although such ECM components as elastin and collagens are known to regulate VSMC tone, VSMC stiffness is emerging as an important determinant of arterial stiffness and tone [14–16]. In addition, VSMC conversion to osteo/chondrogenic phenotype and the subsequent impact on the synthesis of ECM components and cell-ECM interactions can drive arterial stiffening [5].

VSMCs represent the major structural constituents of the arterial wall. VSMCs exhibit the phenotypic plasticity, switching from the physiologically differentiated/contractile phenotype to a de-differentiated/synthetic state upon various pathological stimuli [17, 18]. This process underlies the pathogenesis of most prominent vascular diseases. In contrast to the well-established role of VSMC phenotypic switch in vascular diseases, our knowledge of this process in response to arterial aging has yet to be precisely elucidated. For example, it remains unclear with respect to the occurrence and contribution of VSMC phenotypic switch to arterial stiffness and vascular aging.

Our current knowledge of the molecular underpinnings of age-related phenotypes in the vessel wall is insufficient. A better elucidation and comparison of the genome-wide expression profile of young versus old blood vessels is critical to identify appropriate countermeasures against age-related arterial dysfunction. To that end, we performed bulk RNA sequencing (RNA-seq) on aortae of healthy young (14.5 weeks) and old (92.4 weeks) C57BL/6 mice and performed bioinformatics analysis. We showed that the aged aorta displayed an inflammatory environment with attenuated expression of ECM component genes, especially those for collagen fibril organization. Importantly, we uncovered a significant reduction in the expression of genes involved in protein folding control, stress response, as well as dysregulated circadian core clock genes in old aortae. These molecular alterations may collectively contribute to the development of age-related CVDs. To our knowledge, such transcriptomic analyses have yet to be reported in mice, which represent the foremost mammalian model for studying human health and disease. Our results provide a comprehensive dataset for the investigation of vascular aging, and highlight protein folding mediators and circadian pacemakers as novel targets for the prevention and treatment of age-associated alterations of the vasculature.

## RESULTS

### RNA-seq reveals high divergence of transcriptome profiles between young and old aortae

**Figure 1:**
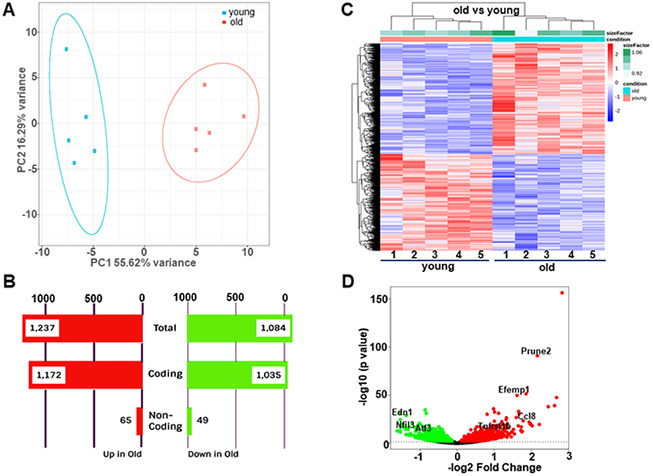
**Differential gene expression between young (14.5 weeks) and old (92.4 weeks) mouse aortae.** (**A**) Principal component analysis (PCA) showing distinct separation of young and old groups. (**B**) Number of differentially expressed transcripts in young versus old aortae. (**C**) Heatmap of hierarchical clustering analysis based on the log2 transformation of the normalized count data. (**D**) Volcano plots showing -log10 (p value) versus -log2 (fold change) of normalized counts between young and old groups. Each dot represented a single transcript. Red dots denoted significant upregulated genes whereas green dots denoted significant downregulated genes (adjusted p<0.05). Several most dramatically altered genes were marked.

We performed RNA-seq analysis on 5 biological replicates of male aortae from each of the young (age 14.5 weeks) and old (age 92.4 weeks) groups. These old mice displayed typical aging phenotype evidenced by decreased Doppler aortic flow velocity (Supplementary Figure 1A) and left ventricular ejection fraction (EF) value (data not shown), evident fibrosis in carotid arteries (Supplementary Figure 1B), as well as induced expression of senescence marker proteins (p21 and p53) in aortae (Supplementary Figure 1C). We obtained approximately 25 million reads per sample (FastP). ~90% of all reads were mapped to the reference mouse genome (mg38+gencode M22 Annotation, STAR). Differences related to sequencing features were not found between two groups. The principle component analysis (PCA) showed that these samples were tightly clustered within each group and there was a clear transcriptome difference between groups (Figure 1A). Next, we conducted differential expression analysis (DEA), with an adjusted p-value threshold of 0.05 on each set of raw expression measures. Overall, there were 2,321 significantly differentially expressed transcripts, which represented 8.3% of the total number of detected transcripts. 1,084 (46.7%) transcripts were downregulated while 1,237 (53.3%) transcripts were upregulated in the aged aorta. More than 90% of differentially expressed genes were found to be protein coding genes, whereas differentially expressed non-coding genes, including long noncoding RNAs (lncRNAs), small ncRNA (microRNA, small nuclear RNA, and small nucleolar RNA), and pseudogenes only accounted for ~5% (Figure 1B). Hierarchical clustering analysis based on the regularized log transformation of the normalized count data showed that all samples clustered by age, indicating that aortic cells from young and old groups have distinct gene expression profiles (Figure 1C). In order to visualize the magnitude of the fold-changes revealed between the two groups, we generated a volcano plot. Genes with adjusted p<0.05 were colored either green or red according to the direction of the fold-change. A line was drawn at the unadjusted p-value of 0.05 for reference. We found that the most significantly downregulated genes in aged aortae included *Edn1*, a potent vasoconstrictor [22], *Nfil3*, a key regulator of circadian rhythm [23], and *Atf3*, a stress responsive transcription factor [24]. The most significantly upregulated genes in aged aortae included *Tnfrsf11b*, a regulator of osteoclast development and risk factor for CVDs [25, 26], and *Ccl8,* a monocyte chemoattractant protein (MCP) family member driving inflammation [27]. In addition, several tumor suppressor genes such as *Prune2* [28] and *Efemp1* [29] were also induced in the old aorta (Figure 1D). Taken together, these results illustrate that substantial numbers of genes are regulated in the aorta in response to aging.

### Functional characterization of gene expression changes between young and old aortae

To identify molecular functions of the aortic transcriptome altered by age, we performed Gene Ontology (GO) enrichment analysis using biological process terms and KEGG pathway analysis. Genes with adjusted p<0.05 and abs (log2FoldChange) > 0 were submitted to Enrichr to identify significantly enriched pathways and transcription factors. Top 30 upregulated and downregulated GO categories/KEGG pathways were presented in Tables 1 and 2, respectively.

**Table 1:** Top 30 upregulated GO Terms and KEGG pathways.

**GO_Biological_Process_2018**			
**Terms**	**Overlap**	**P.value**	**Adjusted. P.value**
cytokine-mediated signaling pathway	87/634	1.71E-12	8.70E-09
cellular response to cytokine stimulus	65/457	2.65E-10	6.77E-07
neutrophil degranulation	64/480	5.07E-09	8.62E-06
neutrophil activation involved in immune response	64/484	7.04E-09	8.98E-06
neutrophil mediated immunity	64/488	9.72E-09	9.92E-06
cellular response to type I interferon	18/66	6.20E-08	5.27E-05
type I interferon signaling pathway	18/66	6.20E-08	4.52E-05
inflammatory response	39/253	1.21E-07	7.71E-05
regulation of small GTPase mediated signal transduction	25/141	1.67E-06	0.000949
positive regulation of phagocytosis	12/40	3.29E-06	0.001676
transmembrane receptor protein tyrosine kinase signaling pathway	48/397	6.80E-06	0.003154
regulation of B cell proliferation	12/44	9.85E-06	0.004188
enzyme linked receptor protein signaling pathway	21/121	1.56E-05	0.006121
T cell activation	17/89	2.77E-05	0.010087
positive regulation of lymphocyte proliferation	15/72	2.79E-05	0.009504
granulocyte chemotaxis	13/57	3.46E-05	0.011035
regulation of B cell receptor signaling pathway	6/17	3.82E-05	0.011464
actin filament bundle organization	10/36	4.56E-05	0.012939
positive regulation of B cell proliferation	9/31	7.49E-05	0.020126
actin filament bundle assembly	10/38	7.58E-05	0.019338
neutrophil chemotaxis	12/54	9.02E-05	0.021916
positive regulation of interferon-beta production	8/27	0.000161	0.037378
neutrophil migration	12/59	0.000221	0.049054
positive regulation of superoxide anion generation	5/11	0.000303	0.064391
regulated exocytosis	21/149	0.000341	0.069578
macrophage activation	6/17	0.000379	0.074309
positive regulation of apoptotic cell clearance	4/7	0.000438	0.082819
microglial cell activation	4/7	0.000438	0.079861
regulation of antigen receptor-mediated signaling pathway	5/12	0.000493	0.086706
T cell differentiation	9/39	0.000503	0.085637
**KEGG_2019_Mouse**			
**Pathways**	**Overlap**	**P.value**	**Adjusted. P.value**
Phagosome	39/180	4.34E-12	1.31E-09
Tuberculosis	34/178	3.39E-09	5.14E-07
Staphylococcus aureus infection	22/95	5.65E-08	5.70E-06
Osteoclast differentiation	25/128	2.53E-07	1.92E-05
Leishmaniasis	17/67	4.48E-07	2.71E-05
Measles	25/144	2.49E-06	0.000126
Hematopoietic cell lineage	19/94	4.00E-06	0.000173
Chagas disease (American trypanosomiasis)	20/103	4.30E-06	0.000163
Complement and coagulation cascades	18/88	5.98E-06	0.000201
Chemokine signaling pathway	29/197	1.22E-05	0.000368
Rap1 signaling pathway	30/209	1.43E-05	0.000395
C-type lectin receptor signaling pathway	20/112	1.60E-05	0.000404
Cell adhesion molecules (CAMs)	26/170	1.71E-05	0.000398
Ras signaling pathway	32/233	1.88E-05	0.000408
Fc gamma R-mediated phagocytosis	17/87	2.03E-05	0.00041
Cytokine-cytokine receptor interaction	37/292	2.71E-05	0.000514
Regulation of actin cytoskeleton	30/217	2.99E-05	0.000532
Natural killer cell mediated cytotoxicity	20/118	3.51E-05	0.000592
Pertussis	15/76	5.44E-05	0.000868
Viral myocarditis	16/87	7.62E-05	0.001155
Leukocyte transendothelial migration	19/115	7.82E-05	0.001128
B cell receptor signaling pathway	14/72	0.000113	0.00156
T cell receptor signaling pathway	17/101	0.000145	0.001907
Epstein-Barr virus infection	29/229	0.000195	0.00246
Primary immunodeficiency	9/36	0.000264	0.003205
Rheumatoid arthritis	14/84	0.000602	0.007017
Ferroptosis	9/40	0.000614	0.006895
Influenza A	22/168	0.000699	0.00756
Malaria	10/49	0.000708	0.007402
Fc epsilon RI signaling pathway	12/68	0.000861	0.008693

**Table 2:** Top 30 downregulated GO terms and KEGG pathways.

**GO_Biological_Process_2018**			
**Terms**	**Overlap**	**P.value**	**Adjusted. P.value**
extracellular matrix organization	41/230	1.36E-11	6.95E-08
response to unfolded protein	16/44	4.92E-10	1.25E-06
substrate adhesion-dependent cell spreading	13/33	6.73E-09	1.15E-05
cell morphogenesis involved in differentiation	15/55	1.43E-07	0.000182
positive regulation of cell differentiation	30/195	2.28E-07	0.000233
sprouting angiogenesis	13/45	4.67E-07	0.000397
chaperone mediated protein folding requiring cofactor	9/25	3.62E-06	0.002642
striated muscle cell development	8/20	5.09E-06	0.003249
negative regulation of protein kinase activity	19/110	6.64E-06	0.003766
positive regulation of angiogenesis	18/104	1.12E-05	0.005716
positive regulation of vasculature development	18/105	1.28E-05	0.005959
negative regulation of protein phosphorylation	25/179	1.31E-05	0.005569
chaperone-mediated protein complex assembly	7/17	1.62E-05	0.006365
regulation of inclusion body assembly	6/12	1.74E-05	0.006348
regulation of cellular response to heat	15/79	1.91E-05	0.006488
collagen fibril organization	9/30	1.98E-05	0.00633
positive regulation of ossification	12/53	2.04E-05	0.006134
'de novo' posttranslational protein folding	9/31	2.66E-05	0.007549
actomyosin structure organization	14/72	2.71E-05	0.007292
regulation of epithelial cell proliferation	14/73	3.19E-05	0.008148
cell-cell junction organization	14/73	3.19E-05	0.00776
negative regulation of kinase activity	16/92	3.19E-05	0.00741
regulation of cellular response to stress	17/105	4.74E-05	0.010523
neuron migration	10/41	5.04E-05	0.010721
protein localization to membrane	22/161	5.94E-05	0.012119
cell junction assembly	10/60	7.55E-05	0.014819
regulation of osteoblast differentiation	13/70	8.60E-05	0.016256
glycosaminoglycan biosynthetic process	16/100	9.08E-05	0.016548
negative regulation of inclusion body assembly	5/10	9.29E-05	0.016344
positive regulation of cardiac muscle cell differentiation	5/10	9.29E-05	0.015799
**KEGG_2019_Mouse**			
**Pathways**	**Overlap**	**P.value**	**Adjusted. P.value**
ECM-receptor interaction	22/83	3.31E-10	1.00E-07
Focal adhesion	32/199	3.14E-08	4.76E-06
Axon guidance	29/180	1.32E-07	1.33E-05
Protein digestion and absorption	17/90	5.82E-06	0.000441
Longevity regulating pathway	18/102	8.47E-06	0.000513
Hippo signaling pathway	23/159	1.61E-05	0.000815
PI3K-Akt signaling pathway	39/357	2.59E-05	0.00112
Fluid shear stress and atherosclerosis	21/143	2.95E-05	0.001118
Signaling pathways regulating pluripotency of stem cells	19/137	0.000152	0.005105
Thyroid hormone signaling pathway	17/115	0.000152	0.004596
Parathyroid hormone synthesis, secretion and action	16/107	0.000205	0.005649
Proteoglycans in cancer	24/203	0.000277	0.006997
AGE-RAGE signaling pathway in diabetic complications	15/101	0.000346	0.008056
Glucagon signaling pathway	15/102	0.000385	0.008339
Wnt signaling pathway	20/160	0.000422	0.008521
Amoebiasis	15/106	0.000585	0.011087
MAPK signaling pathway	30/294	0.000675	0.012032
Hypertrophic cardiomyopathy (HCM)	13/86	0.000708	0.011915
Relaxin signaling pathway	17/131	0.000723	0.011526
Estrogen signaling pathway	17/134	0.000936	0.014178
cGMP-PKG signaling pathway	20/172	0.001063	0.015334
Platelet activation	16/125	0.001187	0.016343
TGF-beta signaling pathway	13/91	0.001215	0.016008
Protein processing in endoplasmic reticulum	19/163	0.00136	0.017171
Pantothenate and CoA biosynthesis	5/18	0.002195	0.026604
Adrenergic signaling in cardiomyocytes	17/148	0.002774	0.032324
Dilated cardiomyopathy (DCM)	12/90	0.003331	0.037377
AMPK signaling pathway	15/126	0.003405	0.036845
Amphetamine addiction	10/68	0.00348	0.036358
Insulin signaling pathway	16/139	0.003563	0.035982

**Figure 2:**
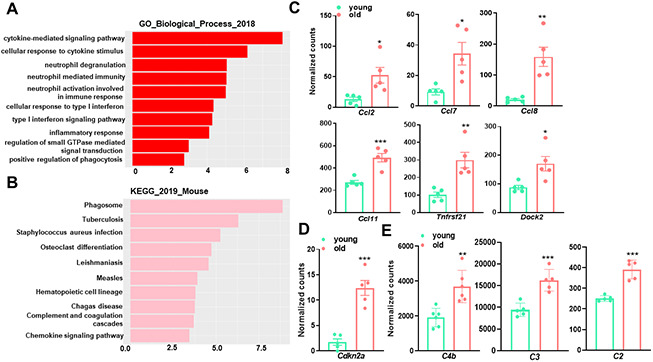
**Gene Ontology (GO) term enrichment and KEGG pathway analysis for upregulated transcripts in young and old mouse aortae.** (**A**) The top 10 enriched GO biological process terms in the transcripts upregulated in old mouse aortae (adjusted p<0.05 and abs (log2FoldChange) > 0). Individual GO terms were sorted by adjusted p values. (**B**) The top 10 enriched KEGG pathways in the transcripts upregulated in old mouse aortae (adjusted p<0.05 and abs (log2FoldChange) > 0). Individual pathways were sorted by adjusted p values. (**C**–**E**) Normalized counts of significantly induced genes in old aortae compared with young counterparts, including proinflammatory genes (**C**), senescence marker (**D**), and complement system components (**E**). Data are presented as a scatterplot of individual points with mean±SD, n=5. *p < 0.05, **p < 0.01, ***p < 0.001 compared to young aortae, unpaired two-tailed Student's t-test.

As shown in Figure 2A, the majority of upregulated pathways in aged aortae were associated with immune activity and inflammation, such as cytokine-mediated signaling pathway (GO:0019221), cellular response to cytokine stimulus (GO:0071345), and neutrophil activation involved in immune response (GO:0002283). The major immune response pathway, type I interferon (IFN) signaling pathway was also significantly induced. Examination of important inflammatory mediators (cytokines, chemokines, and signaling proteins) revealed significant increases in gene expression with aging (Figure 2C). In accord with GO enrichment analysis, tuberculosis, which is an infectious disease closely related to type I IFN [30], was among the top enriched upregulated KEGG pathways (Figure 2B). These results are consistent with previous findings that chronic low-grade inflammation occurs in aged arteries, which could trigger vascular cell senescence and contribute to age-related impairment of vascular function. As expected, *Cdkn2a* (p16^INK4a^), one of the main cyclin-dependent kinase inhibitors (CDKIs) driving the cell cycle arrest in senescence and a widely accepted specific marker for senescence [31], displayed more than 7-fold increase in the aged aorta (Figure 2D). This data is in support of previous studies demonstrating that p16^INK4a^ transcriptional activation is a hallmark of senescence in vivo [32–34].

Additionally, both GO enrichment and KEGG pathway analysis uncovered a significant difference between young and old mouse aortae for genes participating in phagocytosis regulation (Figure 2A, 2B), suggesting an increased capacity to engulf cellular debris or apoptotic cells in the aged aorta. Consistently, C4b, C2, and C3, which are central parts of the complement system enhancing the ability of phagocytic cells to clear microbes and damaged cells [35], were higher in old aortae (Figure 2E). The upregulation of phagocytosis and apoptotic cell clearance may be a feedback response to cellular damage triggered by aging.

**Figure 3:**
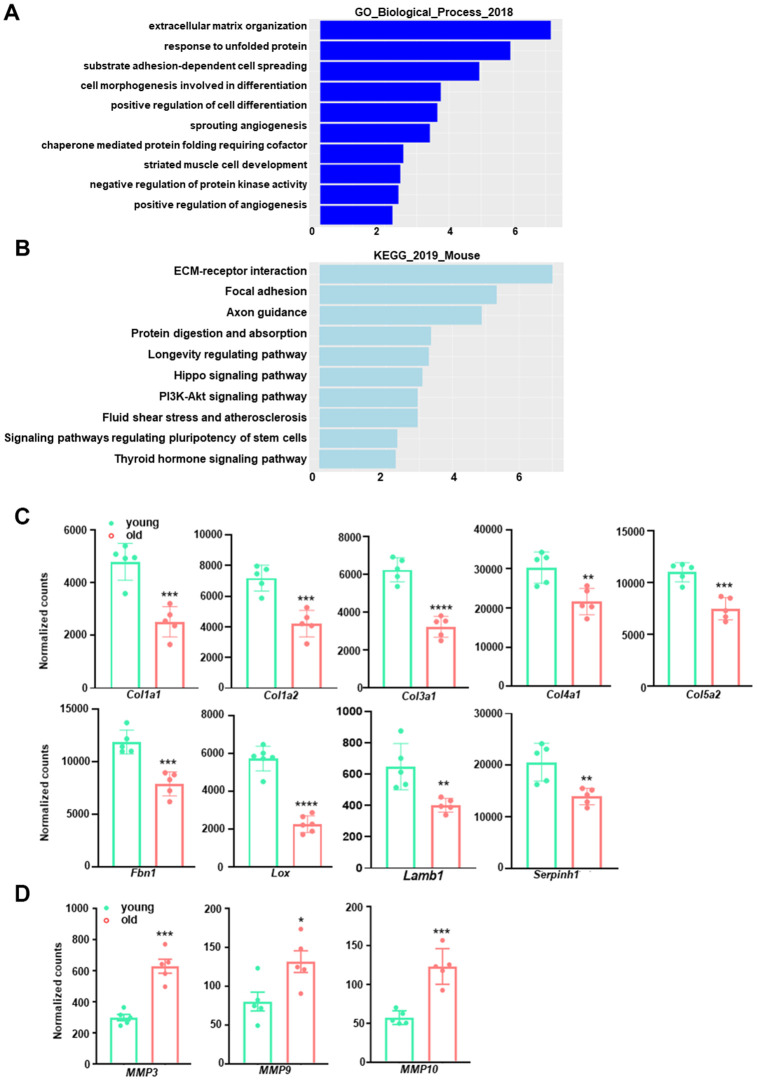
**GO term enrichment and KEGG pathway analysis for downregulated transcripts in young and old mouse aortae.** (**A**) The top 10 enriched GO biological process terms in the transcripts downregulated in old mouse aortae (adjusted p<0.05 and abs (log2FoldChange) > 0). Individual GO terms were sorted by adjusted p values. (**B**) The top 10 enriched KEGG pathways in the transcripts downregulated in old mouse aortae (adjusted p<0.05 and abs (log2FoldChange) > 0). Individual pathways were sorted by adjusted p values. (**C**, **D**) Normalized counts of significantly reduced genes in old aortae compared with young counterparts, including important extracellular matrix (ECM) components or regulators (**C**) and matrix metalloproteinases (MMPs) (**D**) Data are presented as a scatterplot of individual points with mean±SD, n=5. *p < 0.05, **p < 0.01, ***p < 0.001, ****p < 0.0001 compared to young aortae, unpaired two-tailed Student's t-test.

Among the top 10 downregulated pathways in the aged aorta, ECM organization (GO:0030198) was the most significantly altered (Figure 3A), wherein 41 out of 230 genes were downregulated. A similar result was obtained from KEGG pathway analysis, which identified 22 out of 83 downregulated genes in the ECM-receptor interaction pathway (Figure 3B). We further assessed the expression of representative genes involved in ECM and collagen fibril organization. Aging caused a reduction in transcriptional levels of multiple collagen genes, including *Col1a1*, *Col1a2*, *Col3a1*, *Col4a1*, and *Col5a2*, which are abundantly expressed in the vasculature (Figure 3C). Other ECM components or regulators, including *Fbn1*, *Lox*, *Lamb1*, and *Serpinh1*, also exhibited downregulation with aging (Figure 3C). The related GO biological process terms and KEGG pathways, as well as the involved differentially expressed genes, are presented in Table 3. This observation is consistent with a previous study in the aorta of aging rats [36] and the microarray analysis in the aorta of aging monkeys [37], suggesting that differences in the expression of key ECM genes can directly account for the impairment of collagen/elastin crosslinking, resulting in age-related large artery stiffening. Moreover, several well-known matrix metalloproteinase (MMP) genes, including *Mmp3*, *Mmp9*, and *Mmp10*, were elevated in aged aortae (Figure 3D). This increment of MMPs together with the decrement of ECM biosynthesis would compromise the structural integrity of the vasculature, thus contributing to adverse age-associated vascular remodeling.

**Table 3:** Downregulated GO biological process terms and KEGG pathways involving ECM and Collagen Fibril Organization in old aortae.

**Downregulated genes of GO terms of ECM and collagen fibril organization**
Extracellular matrix organization (GO:0030198)	SPARC, COL16A1, ELN, ITGB3, LAMA4, ICAM2, NOXO1, LAMC1, NID2, FBLN5, LOXL2, ADAMTS4, SCUBE3, SH3PXD2A, SERPINH1, ITGA4, LAMB1, NPNT, HSPG2, COL1A1, MFAP5, COL3A1, COL1A2, MMP16, COL4A2, LOX, COL5A1, OPTC, COL4A1, COL5A3, COL6A2, PXDN, COL5A2, COL6A1, COL4A6, ITGA8, PECAM1, COL4A5, AGRN, CD44, FBN1
Collagen fibril organization (GO:0030199)	COL1A1, COL3A1, COL1A2, COL5A1, LOX, COL5A3, COL5A2, SERPINH1, LOXL2
**Downregulated genes of KEGG pathways of ECM receptor interaction and focal adhesion**	
ECM receptor interaction (KEGG_2019_Mouse)	SDC4 ITGB3 LAMA4 GP1BA LAMB1 LAMC1 NPNT HSPG2 COL1A1 COL1A2 COL4A2 COL4A1 COL6A2 COL6A1 CHAD ITGA8 COL4A6 COL4A5 SDC1 AGRN CD44
Focal adhesion (KEGG_2019_Mouse)	GSK3B ITGB3 LAMA4 ILK PIK3CB LAMC1 ACTB MYL12A CCND2 CHAD PAK4 VASP JUN PPP1R12A CAV3 ITGA4 CAV1 LAMB1 IGF1 VEGFA COL1A1 COL1A2 COL4A2 COL4A1 COL6A2 COL6A1 ZYX COL4A6 ITGA8 COL4A5 RAF1

### Pathways related to protein folding control and stress response are predominantly down-regulated in old aortae

**Figure 4:**
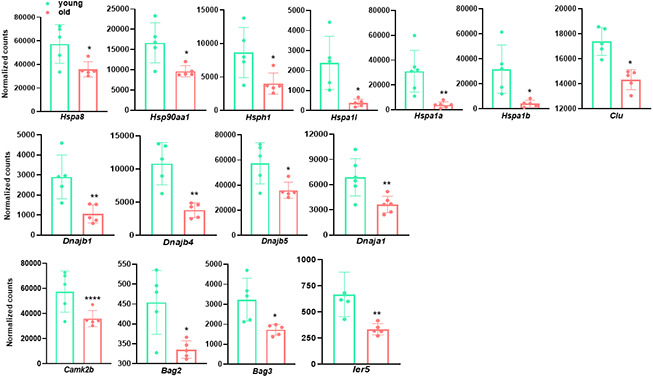
**Genes involved in protein folding process and stress response are decreased in old aortae.** Normalized counts of significantly differentially expressed genes involved in protein folding process and stress response in mouse aortae. Data are presented as a scatterplot of individual points with mean±SD, n=5. *p < 0.05, **p < 0.01, ***p < 0.001, ****p < 0.0001 compared to young aortae, unpaired two-tailed Student's t-test.

In addition to the cluster of genes involved in ECM organization, we observed that “response to unfolded protein (GO:0006986)” and “chaperone mediated protein folding requiring cofactor (GO:0051085)” were among the top 10 enriched downregulated GO terms in the aged aorta (Figure 3A). Several other pathways associated with protein folding process such as “chaperone-mediated protein complex assembly (GO:0051131)” and “'de novo' posttranslational protein folding (GO:0051084)” also displayed significant downregulation (Tables 2, 4). These pathways consisted of numerous differentially expressed genes controlling protein folding, such as heat shock protein family members (e.g. *Hspa8, Hsp90aa1, Hspa1a*), Dnaj heat shock protein family (Hsp40) members (e.g. *Dnaja1, Dnajb1*) and clusterin (*Clu*) (Figure 4). These findings indicate a functional deterioration in protein folding control in the old aorta, particularly chaperone activities, which has been reported as one of the age-related molecular alterations in the vasculature [1, 38, 39].

**Table 4:** Downregulated GO biological terms and KEGG pathways involving protein folding and stress response in old aortae.

**Downregulated genes of GO terms involving protein folding and unfolding process**	
Response to unfolded protein (GO: 0006986)	HSPA8, HSP90AA1, HSP90AB1, HSPA1L, HSPA4, HSPB1, HSPE1, HSPD1, DNAJA1, DNAJB1, HSPH1, DNAJB5, DNAJB4
Chaperone mediated protein folding requiring cofactor (GO: 0051085)	DNAJB1, HSPA8, HSPH1, HSPA1L, HSPA4, PTGES3, HSPE1, HSPA1B, HSPA1A
Chaperone-mediated protein complex assembly (GO: 0051131)	HSP90AA1, HSP90AB1, HSPA4 PTGES3, CLU, HSPD1, HSPA1A
'de novo' posttranslational protein folding (GO:0051084):	DNAJB1, HSPA8, HSPH1, HSPA1L, HSPA4, PTGES3, HSPE1, HSPA1B, HSPA1A
**Downregulated genes of KEGG pathways involving response to stress and heat**	
Regulation of cellular response to heat (GO: 1900034)	CAMK2B, GSK3B, HSPA8, HSP90AA1, HSP90AB1, HSPA1L, HSPB8, PTGES3, DNAJB1, HSPH1, BAG2, BAG3, IER5, HSPA1B, HSPA1A
Regulation of cellular response to stress (GO: 0080135)	CAMK2B, HSPA8, GSK3B, HSP90AA1, HSP90AB1, HSPA1L, HSPB8, PTGES3, NPAS2, ARNTL, DNAJB1, CHCHD2, HSPH1, BAG2, BAG3, HSPA1B, HSPA1A

Reduced protein folding and endoplasmic reticulum (ER) network efficiency with aging results in the accumulation of unfolded proteins, which provokes ER stress and subsequently cellular ER stress response [40]. On a more general basis, aging vessels are confronted by various endogenous and exogenous stressors such as inflammation, oxidative stress, and DNA damage. Therefore, a notably impaired ability of stress response may be a major determinant for the development of age-related vascular pathologies [1]. Consistent with this notion, we found that the regulation of cellular response to heat (GO:1900034, adjusted p =1.91E-05) and stress (GO:0080135, adjusted p = 4.74E-05) were significantly downregulated in aged aortae (Table 4). Reduced expression of several representative genes implicated in cellular response to stress, including *Camk2b, Bag2, Bag3*, *and Ier5,* is shown in Figure 4.

### Circadian rhythm genes are differentially expressed in young and old aortae

**Figure 5:**
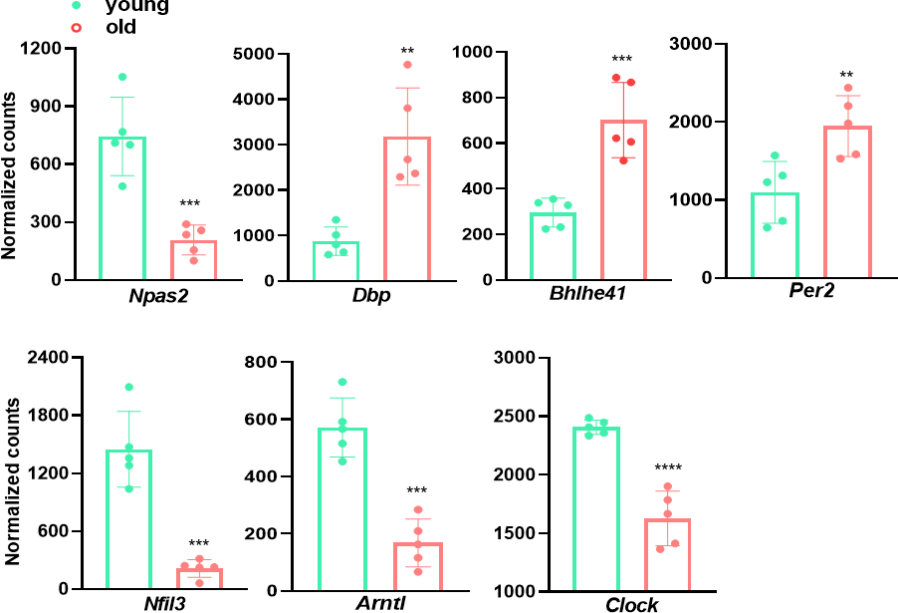
**Circadian rhythm genes are differentially expressed in young and old aortae.** Normalized counts of significantly differentially expressed molecular clock genes in mouse aortae. Data are presented as a scatterplot of individual points with mean±SD, n=5. *p < 0.05, **p < 0.01, ***p < 0.001, ****p < 0.0001 compared to young aortae, unpaired two-tailed Student's t-test.

It has become increasingly appreciated that circadian rhythm disruption precipitates vascular dysfunction [41, 42]. For example, dysregulated expression of circadian clock components results in dysregulation of inflammatory process and ECM dynamics, which are key pathophysiological factors underlying CVDs [42]. To assess the impact of aging on aortic circadian rhythm, we examined the expression of multiple molecular clock genes (Figure 5). *Arntl* (brain-and-muscle ARNT-like protein, also called BMAL1) and *Clock* (circadian locomotor output cycle kaput), the two core transcription factors controlling the transcriptional-translational feedback loop (TTFL) of mammalian clock, exhibited a ~50% and ~30% decrease in old aortae, respectively. Other downregulated clock genes were *Nfil3* (repressor of *Per* transcriptional activity) and *Npas2* (neuronal PAS domain protein 2, *Clock* paralogue), concurring with an upregulation of negative feedback loop components, including *Dbp* (activator of *Per1* transcription), *Bhlhe41* (Basic Helix-Loop-Helix Family Member E41), and *Per2* (repressor of BMAL1/CLOCK complex transcriptional activity) (Figure 5). Altogether, our analysis shows that the transcriptional level of circadian rhythm regulators differs between young and old aortae, supporting the concept that altered circadian rhythm couples with advancing age.

### qRT-PCR validation of representative genes for major terms/pathways regulated by aging

**Figure 6:**
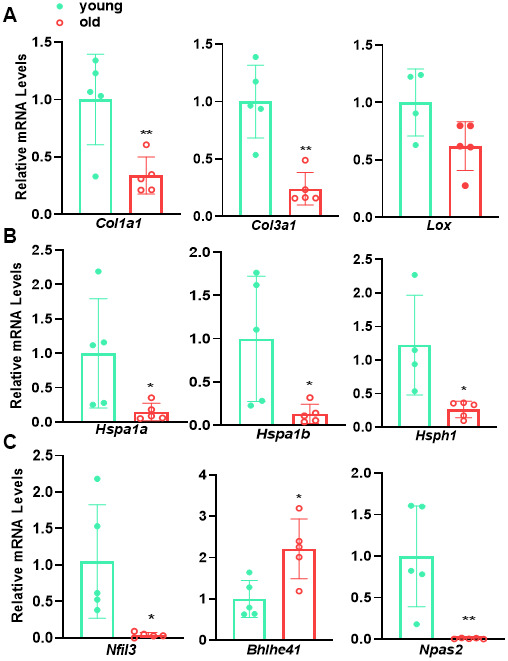
**qRT-PCR validation of representative genes for major age-regulated terms/pathways.** qRT-PCR analysis for the representative genes involving in (**A**) ECM organization, (**B**) protein folding and (**C**) circadian rhythm in young versus old mouse aortae. Data are presented as a scatterplot of individual points with mean±SD, reflecting fold changes over young aortae (set to 1), n=5. *p < 0.05, **p < 0.01, unpaired two-tailed Student's t-test.

To validate age-regulated pathways revealed by aforementioned RNA-seq analysis, we performed qRT-PCR to evaluate the expression of representative genes of several major pathways, including *Col1a1*, *Col3a1*, and *Lox* for ECM organization (Figure 6A), *Hsp1a*, *Hsp1b*, and *Hsph1* for protein folding control (Figure 6B), as well as *Nfil3*, *Bhlhe41*, and *Npas2* for circadian rhythm (Figure 6C). qRT-PCR showed that the expression of these representative genes exhibited similar alterations to those derived from RNA-seq analysis. These results confirmed the regulation of aging on ECM organization, protein folding control, and circadian rhythm pathways revealed by RNA-seq.

### The differentiated VSMC phenotype is retained in aged aortae

**Figure 7:**
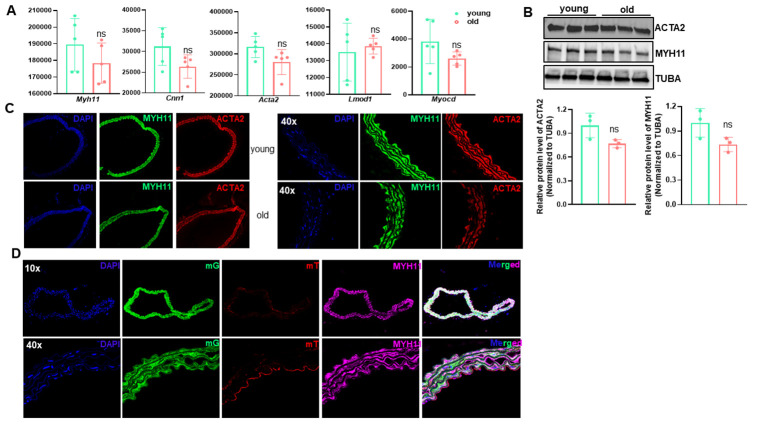
**The contractile vascular smooth muscle cell (VSMC) phenotype is retained in old aortae.** (**A**) Normalized counts of SMC marker genes in young and old mouse aortae. Data are presented as a scatterplot of individual points with mean±SD, n=5. ns, not significant compared to young aortae, unpaired two-tailed Student's t-test. (**B**) Western blotting for the indicated SMC marker proteins in total protein lysates of young and old mouse aortae and its quantitation (n=3). (**C**) Representative confocal microscopy images of immunofluorescence staining for MYH11 and ACTA2 in aortae of young and old C57/BL6 mice. (**D**) Representative confocal microscopy images of immunofluorescence staining for MYH11 and ACTA2 together with fluorescence of membrane Tomato (mT, Red) and membrane GFP (mG, Green) in young and old Myh11-Cre-ERT/mTmG reporter mice. Mice (14 wks) were injected with tamoxifen (TMX) for 5 consecutive days (n=4) and aortae were isolated at the age of 54 wks. Upon TMX induction, all mature SMCs were labeled by mG, whereas other cell types were labeled by mT.

VSMC phenotypic shifting from the quiescent differentiated phenotype to a synthetic dedifferentiated state underlies the pathogenesis of various vascular diseases, including restenosis, atherosclerosis, aneurysm formation, and AVF failure [17–19]. A hallmark of this phenotypic switch is the marked reduction of SMC marker genes. To ascertain whether similar phenotypic switching occurs in arterial aging, we first compared levels of transcripts of SMC markers in young versus old aortae. Bulk RNA-seq revealed a slight and insignificant decrease in SMC marker genes, including *Myh11, Cnn1, Acta2, Lmod1*, and the master regulator of SMC differentiation program, *Myocd* in old aortae (Figure 7A). Further, this subtle downregulation of VSMC marker expression was confirmed by western blot and immunostaining of both MYH11 and ACTA2 (Figure 7B–7C). To determine SMC lineage in old aortae, we sought to take advantage of an established SMC reporter mouse line, Myh11-Cre-ER^T2^/mTmG system [19]. As expected, the medial layer of old aortae was predominately comprised of GFP labeled SMC-derived cells positive for MYH11 and ACTA2 (Figure 7D). These data suggest that though there is a subtle reduction in the expression of SMC markers, these cells maintain the differentiated SMC phenotype.

## DISCUSSION

**Figure 8:**
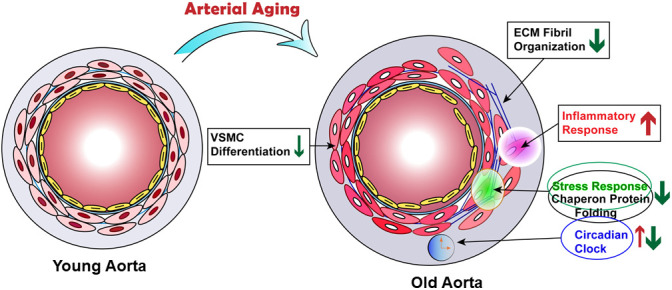
**Proposed model of transcriptome regulated by arterial aging.** Schematic illustrating that arterial aging triggers the activation of inflammatory response and reduction of ECM fibril organization, chaperon-mediated protein folding control and stress response, as well as altered circadian clock.

Advanced medicine and modern lifestyle have resulted in an extended life expectancy. However, this increase in life span is unfortunately accompanied by the occurrence of a number of age-associated diseases such as CVD, neurodegenerative disorder, and cancer [43]. While emerging evidence has pointed to the complexity of arterial aging, a vital process leading to arterial dysfunction and age-related cardiovascular and cerebrovascular diseases, our understanding of what and how diverse pathways intertwine during this process is considerably limited. As such, we performed unbiased genome wide RNA-seq in young and old mouse aortae to dissect the transcriptome regulated by aging. Our results showed that the majority of the upregulated pathways in old aortae related to immune response, including inflammation activation, apoptotic clearance, and phagocytosis. Though excessive collagen accumulation has been reported to contribute to arterial stiffness, ECM organization was the top downregulated pathway in aged aortae. Notably, we found that chaperone-mediated protein folding control and stress response pathways were impaired in aged vessels. We also noticed that circadian core clock genes were differentially expressed in young and old vessels. Finally, transcriptome analysis combined with protein expression validation, as well as SMC lineage tracing showed that SMCs in aged aortae retained the differentiated SMC phenotype, with subtle insignificant downregulation of SMC marker expression (Figure 8). Our results therefore unveiled the critical pathways and involved genes regulated by arterial aging in mice, which provide important insight into the strategies to combat vascular aging and age-associated vascular diseases.

Previous studies have documented a senescence-associated secretory phenotype (SASP) in aged cells, featuring a low grade activation of proinflammatory cytokines/ chemokines, MMPs, and inflammatory mediator gene expression [1, 44]. Consistently, our results revealed an induction of a broad range of proinflammatory genes and cell cycle suppressor p16^INK4A^. Interestingly, we found this proinflammatory phenotype shift was accompanied by a slight downregulation of VSMC differentiation gene program, with an insignificant decrease in VSMC markers and the master regulator MYOCD gene expression. In addition, we did not observe cells positive for either MAC3 (macrophage marker) or CD45 (leukocyte marker) in medial layer of old aortae. This indicates that arterial aging lacks evident “VSMC to macrophage” phenotypic transition and infiltration of proinflammatory cells, both of which occur in vascular disease contexts such as restenosis and atherosclerosis [18, 45, 46]. This is consistent with the notion that aging is a physiological and chronic process attributable to low grade perturbation of multifactorial pathways. A recent study from feed arteries of soleus muscle in rats reported that aged vessels exhibited a decrement in SMC contractility, mainly owing to the impairment of RhoA/ROCK signaling and significant decreased SMα-actin fibers [47]. This is somewhat inconsistent with our current findings wherein genes encoding both cyto-contractile and contraction regulatory proteins (see GSE145972) were insignificantly attenuated. Whether these marginally reduced gene programs hamper vessel contraction of old aortae in mice and humans warrants further investigation.

One notable finding in this study was the downregulation of key pathways controlling protein folding process. These included response to unfolded protein, chaperone mediated protein folding requiring cofactor, chaperone-mediated protein complex assembly, and 'de novo' posttranslational protein folding. This indicates that protein folding control, a critical process of proteostasis, is impaired in aged arteries. This finding is in agreement with previous reports that loss of proteostasis is a major feature of aging and age-associated degenerative diseases [1, 10, 48, 49]. Interestingly, the majority of downregulated genes within these pathways are those encoding heat shock proteins (HSPs), which is consistent with the downregulation of stress and heat shock response pathways revealed by GO analysis. We noticed that the most dramatically reduced genes (>6 fold) were the three HSP70 alternative splicing variants, namely *Hspa1a*, *Hspa1b*, and *Hspa1l*. HSP70 family members have been established as the vital components of the cellular network of molecular chaperones and folding catalysts. Though downregulation of these molecular chaperone components has been demonstrated as an important contributor to aging and age-associated degenerative proteopathies, including polyQ (polyglutamine) disease, Alzheimer’s disease, and coronary artery disease [50, 51], the underlying mechanism remains enigmatic. The reduced expression of HSP family members unveiled in our study implies that this downregulation might occur at the transcriptional level. It is surprising that the established transcription factor for HSP family members, HSFs (a family of heat shock factor (HSF) transcription factors) [52], were not significantly regulated (see GSE145972). This raises the possibility that there might exist unknown regulator(s) of HSP70 gene transcription, which is of great interest to investigate in the future. Beyond protein folding to ensure the correct conformation of protein and therefore avoid protein aggregates, proteostasis is also subjected to the regulation from proteolytic systems, mainly ubiquitin-proteasome and lysosomal autophagy systems to degrade protein aggregates [10, 48, 49, 51]. In contrast to the striking downregulation of protein folding pathways, our results failed to reveal significant changes in ubiquitination and autophagy pathways. Indeed, gene expression of key components of ubiquitination network, such as *Cul3*, *Nae1*, *Mdm2,* and *Ring1*, as well as autophagy machinery, *Sqstm1* and ATG family members, stayed the same in young and old aortae (see GSE145972). Therefore, the decline in protein homeostasis with aging might largely be attributed to the impaired transcription of the molecular chaperones for protein folding control.

Circadian rhythm is pervasive in vascular system. Numerous structural and regulatory genes are oscillated in a circadian rhythmic manner in the blood vessel system, resulting in many critical circadian rhythmic vascular functions, such as vessel wall contraction, leukocyte adhesion, and platelet aggregation [53–55]. In general, circadian rhythm is integrally governed by the core clock genes comprised of two activators, Clock, Bmal1 (ARNTL) and two repressors, Period (Per) and Cryptochrome (Cry). These core circadian components constitute a unique intrinsic TTFL to ensure their own circadian rhythmic gene expression pattern [56, 57]. The association of core clock gene expression with aging is controversial and likely context dependent. Although mounting evidence has stressed the importance of circadian rhythm in vascular homeostasis, no studies thus far have directly connected the circadian clock genes to vascular aging. We found that the transcription activators of circadian clock machinery, *Arntl*, *Clock*, and *Nasp2*, were significantly decreased, whereas the repressors, *Per1/Per2*, and *Bhlhe41* were increased in aged aortae. This reciprocal correlation of molecular clock activator and repressor to vascular aging is consistent with their intrinsic negative feedback required for their 24 hours rhythmic gene expression. Beyond these core clock components, another well-known circadian clock gene, *NfIl3* (E4B4), was also drastically downregulated in aged vessels. It has been reported that NFIL3 represses *Per1/Per2* gene transcription [58]. Therefore, reduced *Nfil3* might contribute to the elevated *Per1/Per2* gene expression in aged vessels revealed in our study. Altogether, these results indicate that the circadian core machinery is tightly regulated by vascular aging. The mechanism underlying aging regulation of circadian clock gene expression is virtually unknown. Nevertheless, a recent report suggests that cellular senescence impairs circadian rhythmicity, which might relate to the decreased ability of senescent cells to transmit nitric oxide, an important circadian signal to their clocks [7]. Interestingly, our study showed that *Nos3* (eNOS), the key enzyme to synthesize nitric oxide in the vasculature wall was downregulated (see GSE145972), which might further attenuate NO signaling for circadian rhythm control. Whether this senescence associated impairment in NO signaling leads to the dysregulated molecular clock gene expression in aged arteries requires further investigation.

The mammalian genome is extensively transcribed, with the majority of transcripts falling into the noncoding class [59, 60]. Careful analysis of noncoding genes from our RNA-seq data showed that only 10,662 noncoding genes, a lower number than protein coding genes (17,349), were detected in our study. The majority of these noncoding genes were of low counts, which is in accordance with the much lower abundance of most long noncoding RNAs (lncRNAs) compared with protein coding genes. Among all detected noncoding genes, only 65 and 49 were upregulated and downregulated, respectively, in aged aortae. We attribute this low number of noncoding transcripts to the insufficient reads (30 million per sample) and selection for polyadenylated (poly(A)) RNA transcripts employed in our RNA-seq. Regardless, the small portion of noncoding genes regulated by arterial aging also suggest that noncoding gene transcription may resist to the low-grade chronic stress derived from physiological aging. This might serve as a potential distinction between physiological aging and vascular pathology, the latter involves the emerging roles of noncoding genes, in particular microRNAs and lncRNAs.

There are two evident limitations in our current study. First, our study was confined to male mice. Emerging evidence suggests gender differences in aging and aging-associated vascular diseases. It will therefore be of high interest to determine if the distinct pathways revealed in male mice could also apply to female mice. Second, the RNA samples used for our bulk RNA-seq were extracted from aortae wherein only periadventitial adipose tissue was removed; it is unclear at this time whether cells other than bonafide SMC (e.g., resident stem cells in vessel wall) contribute to the changes in these pathways. This enigma could be resolved by single cell RNA-seq in concert with lineage tracing reporter system in young versus aged aortae.

In summary, our unbiased study revealed the transcriptome regulated by physiological aging in mouse aortae. In addition to validation of previously well-recognized SASP exhibited in aged aortae, we uncovered a significant downregulation in gene expression of molecular chaperone HSP70 family members, as well as the dysregulated circadian core clock machinery in aged aortae. Our results therefore unveiled critical pathways and involved genes regulated by arterial aging, which will have important implications for strategies to combat vascular aging and age-associated vascular diseases such as atherosclerosis and aneurysm.

## MATERIALS AND METHODS

### Animals

All experiments were performed in compliance with approved protocols of the Institutional Animal Care and Use Committee (IACUC) at Albany Medical College. Both young (2 months) and old (18 months) male C57BL/6 mice were purchased from Jackson laboratory. Both groups were fed with water and standard rodent chow diet for more than one month before sacrifice at the same time for experiments. Young (14.5 weeks) and old (92.4 weeks) mice were sacrificed at the same time followed by tissue isolation for RNA preparation. In order to track VSMCs lineage in old aortae, we bred Myh11-CreER^T2^ (JAX, # 007576) to ROSA mT/mG mice (JAX, # 01979) to generate Myh11-Cre/ER^T2^-mTmG mice. These lineage tracing mice were injected with Tamoxifen intraperitoneally at an early stage (14 weeks) to activate Cre recombinase in mature VSMCs as previously described to specifically label SMCs with membrane green fluorescence (mG) and other cell types with membrane red fluorescence (mT) [19]. These reporter mice were sacrificed at the age of 54 weeks for the indicated lineage tracing study.

### RNA isolation and deep sequencing

Aortae were quickly excised from saline perfused mice and cleaned of periadventitial adipose tissue. Purified aortae (5 aortae/group) were homogenized by a Minilys homogenizer (Bertin Technologies, Rockville, MD, USA) before total RNA extraction using miRNeasy RNA extraction kit (Qiagen, Valenica, CA, USA). RNA integrity was examined with an Experion™ Automated Electrophoresis System by utilizing Experion RNA StdSens analysis kit (Bio-Rad, Hercules, CA, USA). Bulk RNA-seq was conducted by Genomics Research Center at the University of Rochester Medical Center. Detailed information regarding library construction and sequence depth was described previously [20]. Raw data derived from RNA-seq was filtered using FastP-0.20.0 (https://github.com/OpenGene-/fastp). To obtain the high-quality clean reads for subsequent analysis, reads below a length of 35 after read trimming by quality were removed from the sequencing data. Sequence reads were aligned to annotated transcripts on the UCSC Reference Genome (mg38 + gencode M22 Annotation) via STAR-2.7.0f. Subread-1.6.4 feature counts were used to count reads at the gene level. Differentially expressed gene analysis DESeq2-1.22.1 within R-3.5.1 was used to perform data normalization and differential expression analysis with an adjusted p-value threshold of 0.05 on each set of raw expression measures. The ‘lfcShrink’ method was applied to moderate log2 fold-changes for lowly expressed genes. The detailed RNA-seq information of this assay is available in GSE145972 deposited in NIH Gene Expression Omnibus (GEO) database.

### Pathway enrichment analysis

Significant up-regulated and down-regulated genes based on p-adj <0.05 and abs (log2FoldChange) > 0 were submitted to Enrichr (https://amp.pharm.mssm.edu/Enrichr/) to identify significantly enriched pathways and transcription factors.

### Quantitative reverse transcription-polymerase chain reaction (qRT-PCR)

After RNA samples were extracted from mouse aortic tissues, cDNA synthesis was conducted by using iScript cDNA kit (Bio-Rad). qRT-PCR was performed using Universal SYBR Green Supermix (Bio-Rad) and CFX386 Touch™ Real-Time PCR Detection System (Bio-Rad). mRNA levels were expressed relative to the loading control *Gapdh*. Technical duplicates of each sample were examined. PCR primers are included in Supplementary Table 1.

### Immunofluorescence Staining

Freshly harvested mouse aortae were rinsed in cold 1XPBS and immediately fixed in 4% paraformaldehyde at 4 °C for 24 hours followed by OCT processing. 10 μm frozen sections were prepared for immunofluorescence (IF) staining as previously described [21]. For aortae isolated from mTmG reporter mice, we used trypsin-mediated antigen retrieval (REF TA-015–TR; Lab Vision Corporation, Fremont, CA) to retain the natural mTmG fluorescence prior to the immunofluorescence staining. Primary antibodies used were as follows: anti-MYH11 (1:500, Alfa Aesar, BT-562), anti-ACTA2 (1:2000, Sigma, A2547). Fluorescent signals were captured by a confocal microscope and processed by Photoshop (Adobe, San Jose, CA, USA). All images were captured and processed under equivalent conditions.

### Statistical analysis

Statistical analysis was determined by an unpaired two-tailed Student’s t test using GraphPad Prism 6 to compare two groups. Data in graphs were presented as mean ± SD. *p*<0.05 was considered statistically significant. *p*<0.05 and *p*<0.01 were indicated by * and **, respectively, throughout all graphs.

## Supplementary Material

Supplementary Figure 1

Supplementary Table 1
