# Supplementary material for: Transcriptome analysis of mouse aortae reveals multiple novel pathways regulated by aging
Source: Aging (Albany NY). 2020 Aug 15;12(15):15603–23. doi: 10.18632/aging.103652 (PMC7467355; doi:10.18632/aging.103652)
Supplement: Supplementary Figure 1 [file aging-12-103652-s001..pdf]

SUPPLEMENTARY FIGURE

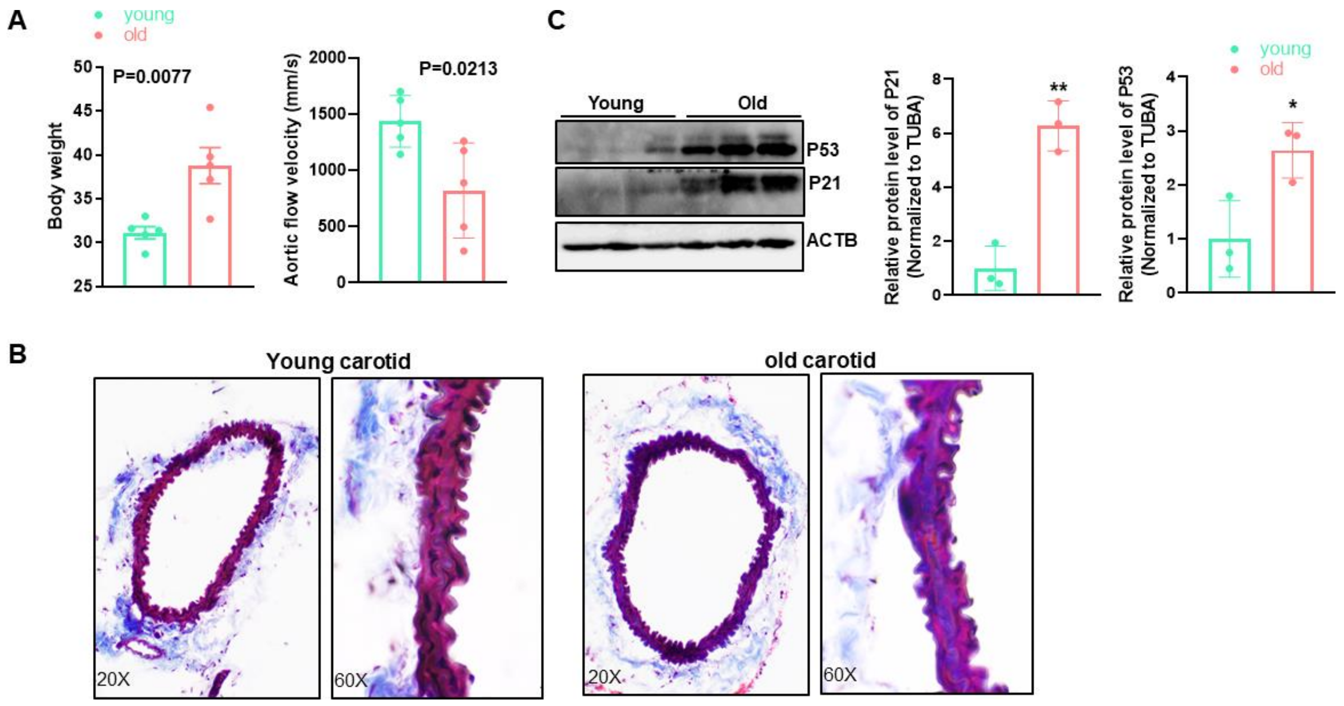

Supplementary Figure 1. Vascular aging phenotype confirmation of the old mice utilized for RNA-seq analysis. (**A**) Body weight and Doppler aortic flow velocity measurements for the young and old mice 2 days before organ isolation for RNA-seq. (**B**) Representative images of trichrome staining for carotid arteries from the same batch of young and old mice utilized for RNA-seq analysis (n=3). (**C**) Western blotting for the indicated senescence marker proteins in total protein lysates of young and old mouse aortas and the quantitation (n=3). *p < 0.05, **p < 0.01, unpaired two-tailed Student's t-test.
