# Supplementary material for: Transcriptome analysis of mouse aortae reveals multiple novel pathways regulated by aging
Source: Aging (Albany NY). 2020 Aug 15;12(15):15603–23. doi: 10.18632/aging.103652 (PMC7467355; doi:10.18632/aging.103652)
Supplement: Supplementary Table 1 [file aging-12-103652-s002..pdf]

SUPPLEMENTARY TABLE

Supplementary Table 1. Sequence information of primers used for qRT-PCR.

Gene	Strand	Primer Sequence	Application(s)
Col1a1	Forward	TTCGTGACCGTGACCTTGAG	qRT-PCR
	Reverse	CCAGGTTGCAGCCTTGGTTA	
Col3a1	Forward	GCCACCTTGGTCAGTCCTAT	qRT-PCR
	Reverse	GAAGCACAGGAGCAGGTGTA	
Lox	Forward	ACGCTGTGACATTCGCTACA	qRT-PCR
	Reverse	TGTCCAAACACCAGGTACGG	
Hspa1a	Forward	TCGAGGAGGTGGATTAGAGG	qRT-PCR
	Reverse	GTCTAGGACTTGATTGCAGGAC	
Hspa1b	Forward	TCGAGGAGGTGGATTAGAGG	qRT-PCR
	Reverse	ACCTTGACAGTAATCGGTGC	
Hspah1	Forward	GCTAGACGTAGGCTCACAGAG	qRT-PCR
	Reverse	GGCTGCAACTCCAATTGTTC	
Nfil3	Forward	CAGGGAGCAGAACCACG	qRT-PCR
	Reverse	CTTAAGGACTTCAGCCTCTCATC	
Bhlhe41	Forward	GTGTAAACCCAAAAGGAGCTTG	qRT-PCR
	Reverse	CAATTTCAGATGTTCGGGCAG	
Npas2	Forward	CCCACTACTACATCACCTACCA	qRT-PCR
	Reverse	GTCTCCTTTCCACTCGAACATC	
